# A Universal Curse

**Published:** 1892-11

**Authors:** 


### A UNIVERSAL CURSE.

It is a notable and significant fact that the drink curse has been acknowledged as one of the chief factors in two great evils which overshadow the civilized world, and that it was also one of the moving causes in a recent event which threatened at one time to plunge two nations into the horrors of war. We refer to the famine in Russia, the ruin and degradation of the natives of Africa, and the quarrel with Chile. In a recent interview with Count Tolstoi, published in the *World*, of this city, the Russian philanthropist distinctly mentions drunkenness of one of three things which have led to the present distress. The other two things are improvidence and despair.

The Russian correspondent of the New York *Times*, and others who have investigated the famine districts, bear testimony to the same facts. The terrible vodka, the Russian drink, is at the bottom of a large part of the misery. As for what rum is doing for the natives of Africa, all the world knows the shameful story. We are rightfully indignant at the hideous cruelties practised by the Arab slave dealers in carrying on their traffic among the poor negroes of the Dark Continent, but it may be doubted whether the rum imported from Christian nations, and largely from our own country, has not been, in recent years, as heavy a curse upon these heathen people as the Arab slave trade.

Slavery may have affected a much larger number of people, but it is not an evil that takes hold of the blood. Rum not only curses those who drink to-day, but it reaches out with deadly and paralyzing grasp upon future generations. We may put an end to slavery by force, but bayonets and cannon will not eliminate the rum poison which we have put into the veins of the African negro. And then as to the Chilean quarrel, it is a generally conceded fact that the riot in Valparaiso, which started all the trouble, had its occasion partly, if not chiefly, in the drunken antics of some American sailors.

And so it is all around. Whichever way we turn in our effort to ameliorate the condition of mankind, whether it be in its social, political, or industrial status, we find the drink curse in the way.—*Christian at Work.*

